# Methyl 2-allyl-4-hydr­oxy-2*H*-1,2-benzothia­zine-3-carboxyl­ate 1,1-dioxide

**DOI:** 10.1107/S160053680904673X

**Published:** 2009-11-14

**Authors:** Muhammad Nadeem Arshad, Muhammad Zia-ur-Rehman, Islam Ullah Khan

**Affiliations:** a Department of Chemistry, Government College University, Lahore 54000, Pakistan; b Applied Chemistry Research Centre, PCSIR Laboratories Complex, Ferozpure Road, Lahore 54600, Pakistan

## Abstract

In the title compound, C_13_H_13_NO_5_S, the thia­zine ring adopts a distorted half-chair conformation. Intra­molecular O—H⋯O and C—H⋯O hydrogen bonds give rise to two six-membered hydrogen bonded rings. In the crystal, mol­ecules are linked through weak inter­molecular C—H⋯O hydrogen bonds, resulting in a zigzag chain lying along the *c* axis.

## Related literature

For the syntheses of related compounds, see: Braun (1923 ▶); Zia-ur-Rehman *et al.* (2005 ▶). For the biological activity of benzothia­zines, see: Zia-ur-Rehman *et al.* (2006 ▶, 2009 ▶). For related structures, see: Arshad *et al.* (2009 ▶); Fabiola *et al.* (1998 ▶); Zia-ur-Rehman *et al.* (2007 ▶).
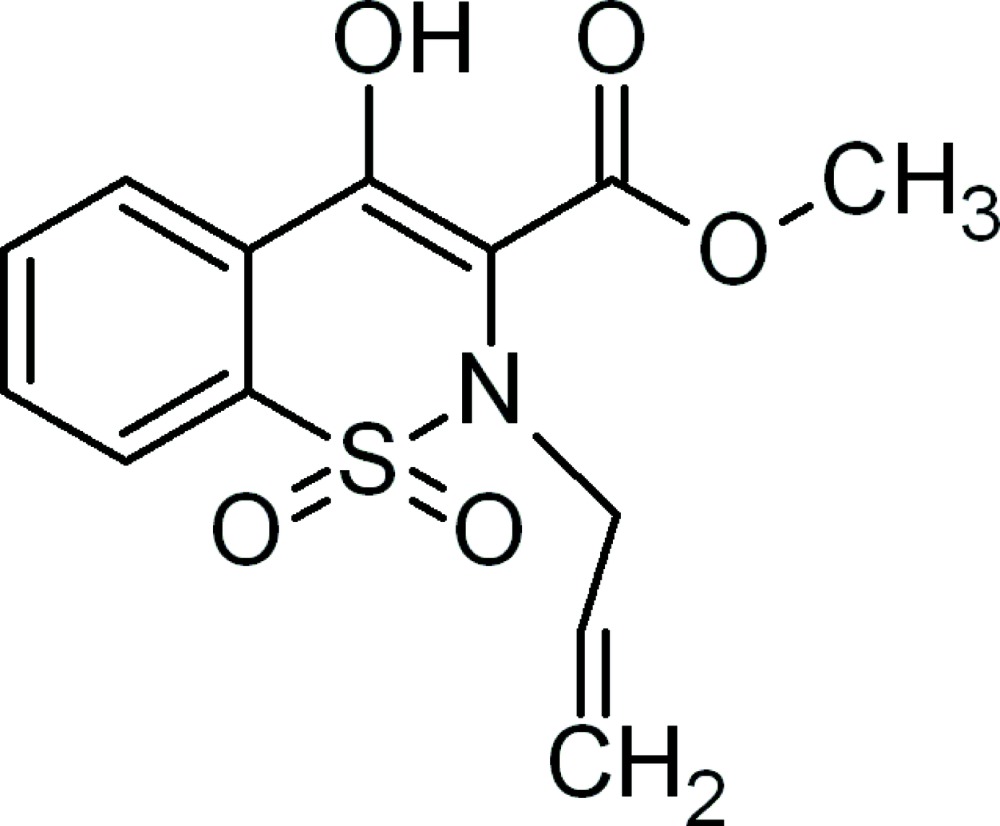


## Experimental

### 

#### Crystal data

C_13_H_13_NO_5_S
*M* *_r_* = 295.30
Orthorhombic, 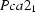

*a* = 12.4289 (10) Å
*b* = 8.3706 (8) Å
*c* = 13.0132 (11) Å
*V* = 1353.9 (2) Å^3^
*Z* = 4
Mo *K*α radiation
μ = 0.26 mm^−1^
*T* = 296 K
0.45 × 0.11 × 0.07 mm

#### Data collection

Bruker APEXII CCD area-detector diffractometer
Absorption correction: multi-scan (**SADABS**; Sheldrick, 1996 ▶) *T*_min_ = 0.893, *T*_max_ = 0.982
8404 measured reflections
2808 independent reflections
1763 reflections with *I* > 2σ(*I*)
*R*_int_ = 0.044

#### Refinement

*R*[*F* ^2^ > 2σ(*F* ^2^)] = 0.057
*wR*(*F* ^2^) = 0.103
*S* = 1.06
2808 reflections
183 parameters
1 restraint
H-atom parameters constrained
Δρ_max_ = 0.18 e Å^−3^
Δρ_min_ = −0.23 e Å^−3^
Absolute structure: Flack (1983 ▶), 1046 Friedel pairs
Flack parameter: 0.07 (11)

### 

Data collection: *APEX2* (Bruker, 2007 ▶); cell refinement: *SAINT* (Bruker, 2007 ▶); data reduction: *SAINT*; program(s) used to solve structure: *SHELXS97* (Sheldrick, 2008 ▶); program(s) used to refine structure: *SHELXL97* (Sheldrick, 2008 ▶); molecular graphics: *PLATON* (Spek, 2009 ▶) and *Mercury* (Macrae *et al.*, 2006 ▶); software used to prepare material for publication: *WinGX* (Farrugia, 1999 ▶) and *PLATON*.

## Supplementary Material

Crystal structure: contains datablocks I, global. DOI: 10.1107/S160053680904673X/is2483sup1.cif

Structure factors: contains datablocks I. DOI: 10.1107/S160053680904673X/is2483Isup2.hkl

Additional supplementary materials:  crystallographic information; 3D view; checkCIF report

## Figures and Tables

**Table 1:** Hydrogen-bond geometry (Å, °)

*D*—H⋯*A*	*D*—H	H⋯*A*	*D*⋯*A*	*D*—H⋯*A*
O3—H3*O*⋯O4	0.82	1.84	2.555 (4)	146
C11—H11*A*⋯O5	0.97	2.50	3.055 (4)	116
C3—H3*A*⋯O1^i^	0.93	2.51	3.406 (6)	163

Symmetry code: (i) 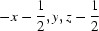.

**Fig. 1.**
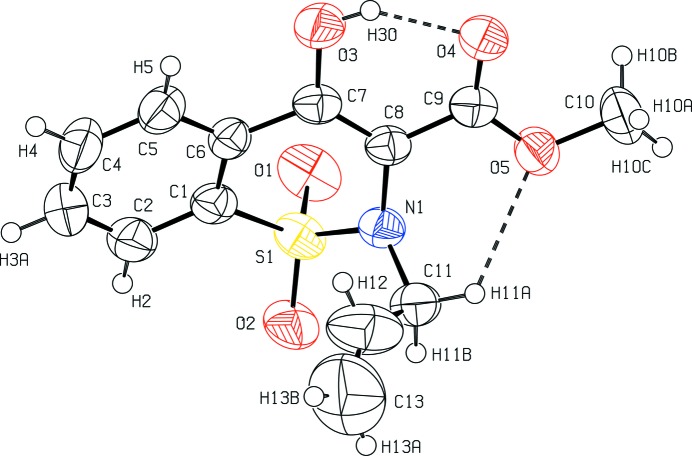
The molecular structure of (I), with displacement ellipsoids at the 50% probability level.

**Fig. 2.**
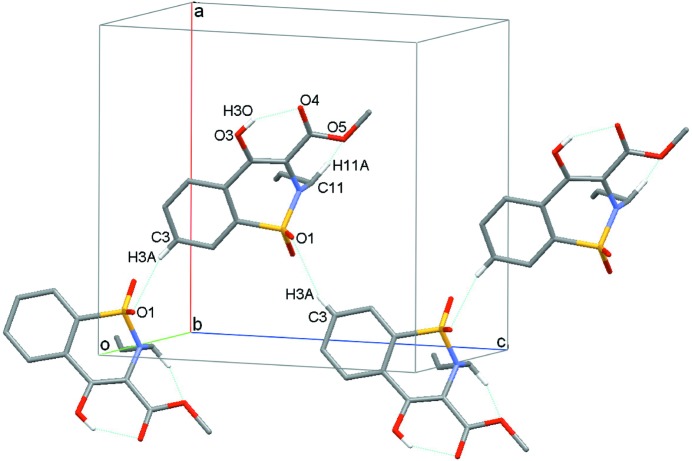
Perspective view of the crystal packing showing O—H···O and C—H···O hydrogen-bonded interactions (dashed lines). H atoms not involved in hydrogen bonding have been omitted for clarity.

## supplementary crystallographic information

### Comment

Benzothiazine1,1-dioxides are familiar for their different type of biological activities (Zia-ur-Rehman *et al.*, 2006) and have been synthesized continuously since the very first synthesis in 1923 (Braun, 1923). In continuation of our work on he synthesis of various bioactive benzothiazines (Zia-ur-Rehman *et al.*, 2005, 2009), we herein report the crystal structure of the title compound (I), thiazine ring exhibits a distorted half-chair conformation with S1/C1/C6/C7 atoms lying in a plane and N1 showing significant departure from the plane due to its pyramidal geometry projecting the allyl group approximately perpendicular to the ring (Fig. 1). Like previously reported crystal structures of various 1,2-benzothiazine 1,1-dioxide derivatives (Arshad *et al.*, 2009; Fabiola *et al.*, 1998; Zia-ur-Rehman *et al.*, 2007), the enolic hydrogen on O3 is involved in intramolecular hydrogen bonding (Table1). In addition, C11—H11A···O5 hydrogen bond gives rise to another six-membered hydrogen ring in the molecule while C7—C8 bond length [1.338 (5) Å] (very close to normal C—C bond; 1.36 Å) indicates a partial double-bond character indicating the dominance of enolic form in the molecule. The C1—S1 bond distance [1.746 (4) Å] is as expected for typical C(*sp*2)—S bond (1.751 Å). Each molecule is linked to neighbouring molecules *via *weak C—H···O=S interactions giving rise to zigzag chains along the *c* axis (Fig. 2).

### Experimental

Allyl iodide (5.04 g, 30.0 mmol) was added drop wise to the mixture of methyl 4-hydroxy-2*H*-1,2-benzothiazine-3-carboxylate-1,1-dioxide (3.83 g, 15.0 mmol), anhydrous potassium carbonate (1.68 g, 30.0 mmol) and dimethylformamide (20 ml) in a round bottom flask. Contents were stirred at room temperature for 7 h under nitrogen atmosphere and poured over ice cooled water (300 ml) resulting in an immediate formation of a white solid, which was filtered and washed with cold water. Single crystals were obtained by re-crystallization from a methanol solution

### Refinement

All hydrogen atoms were positions geometrically and treated as riding on their parent atoms. The following distances were used: methyl C—H = 0.96 Å, methylene C—H = 0.97 Å, aromatic C—H = 0.93 Å and hydroxyl O—H = 0.82 Å. *U*_iso_(H) was set to 1.2*U*_eq_ of the parent atoms or 1.5*U*_eq_ for methyl and hydroxyl groups. Large thermal displacement parameters for the terminal carbon atoms (C12 and C13) are observed but the disorder produce was not resolved.

### Crystal data

**Table:**

C_13_H_13_NO_5_S	*F*(000) = 616
*M**_r_* = 295.30	*D*_x_ = 1.449 Mg m^−^^3^
Orthorhombic, *P**c**a*2_1_	Mo *K*α radiation, λ = 0.71073 Å
Hall symbol: P 2c -2ac	Cell parameters from 1619 reflections
*a* = 12.4289 (10) Å	θ = 3.1–21.7°
*b* = 8.3706 (8) Å	µ = 0.26 mm^−^^1^
*c* = 13.0132 (11) Å	*T* = 296 K
*V* = 1353.9 (2) Å^3^	Needle, colourless
*Z* = 4	0.45 × 0.11 × 0.07 mm

### Data collection

**Table:**

Bruker APEXII CCD area-detector diffractometer	2808 independent reflections
Radiation source: fine-focus sealed tube	1763 reflections with *I* > 2σ(*I*)
graphite	*R*_int_ = 0.044
φ and ω scans	θ_max_ = 28.3°, θ_min_ = 2.9°
Absorption correction: multi-scan (*SADABS*; Sheldrick, 1996)	*h* = −16→16
*T*_min_ = 0.893, *T*_max_ = 0.982	*k* = −11→10
8404 measured reflections	*l* = −17→9

### Refinement

**Table:**

Refinement on *F*^2^	Secondary atom site location: difference Fourier map
Least-squares matrix: full	Hydrogen site location: inferred from neighbouring sites
*R*[*F*^2^ > 2σ(*F*^2^)] = 0.057	H-atom parameters constrained
*wR*(*F*^2^) = 0.103	*w* = 1/[σ^2^(*F*_o_^2^) + (0.0381*P*)^2^ + 0.1044*P*] where *P* = (*F*_o_^2^ + 2*F*_c_^2^)/3
*S* = 1.06	(Δ/σ)_max_ < 0.001
2808 reflections	Δρ_max_ = 0.18 e Å^−^^3^
183 parameters	Δρ_min_ = −0.23 e Å^−^^3^
1 restraint	Absolute structure: Flack (1983), 1046 Friedel pairs
Primary atom site location: structure-invariant direct methods	Flack parameter: 0.07 (11)

### Special details

**Table:**

Geometry. All e.s.d.'s (except the e.s.d. in the dihedral angle between two l.s. planes) are estimated using the full covariance matrix. The cell e.s.d.'s are taken into account individually in the estimation of e.s.d.'s in distances, angles and torsion angles; correlations between e.s.d.'s in cell parameters are only used when they are defined by crystal symmetry. An approximate (isotropic) treatment of cell e.s.d.'s is used for estimating e.s.d.'s involving l.s. planes.
Refinement. Refinement of *F*^2^ against ALL reflections. The weighted *R*-factor *wR* and goodness of fit *S* are based on *F*^2^, conventional *R*-factors *R* are based on *F*, with *F* set to zero for negative *F*^2^. The threshold expression of *F*^2^ > σ(*F*^2^) is used only for calculating *R*-factors(gt) *etc*. and is not relevant to the choice of reflections for refinement. *R*-factors based on *F*^2^ are statistically about twice as large as those based on *F*, and *R*- factors based on ALL data will be even larger.

### Fractional atomic coordinates and isotropic or equivalent isotropic displacement parameters (Å^2^)

**Table:**

	*x*	*y*	*z*	*U*_iso_*/*U*_eq_	
S1	−0.11229 (6)	0.28643 (12)	0.49624 (9)	0.0550 (3)	
O1	−0.12270 (18)	0.1530 (4)	0.5634 (3)	0.0733 (9)	
O2	−0.19486 (15)	0.4041 (3)	0.4937 (3)	0.0714 (8)	
O3	0.18469 (19)	0.0795 (3)	0.4129 (2)	0.0669 (9)	
H3O	0.2303	0.0963	0.4571	0.100*	
O4	0.26461 (18)	0.2122 (3)	0.5724 (2)	0.0658 (8)	
O5	0.16238 (19)	0.3843 (3)	0.6600 (2)	0.0612 (7)	
N1	0.0004 (2)	0.3754 (3)	0.5229 (2)	0.0490 (9)	
C1	−0.0885 (3)	0.2109 (4)	0.3733 (3)	0.0487 (10)	
C2	−0.1708 (3)	0.1997 (5)	0.3013 (4)	0.0647 (12)	
H2	−0.2396	0.2360	0.3168	0.078*	
C3	−0.1488 (4)	0.1344 (5)	0.2074 (4)	0.0737 (13)	
H3A	−0.2034	0.1247	0.1590	0.088*	
C4	−0.0462 (4)	0.0828 (5)	0.1837 (4)	0.0721 (12)	
H4	−0.0319	0.0406	0.1191	0.087*	
C5	0.0348 (3)	0.0935 (5)	0.2553 (3)	0.0602 (12)	
H5	0.1032	0.0564	0.2391	0.072*	
C6	0.0156 (3)	0.1588 (4)	0.3509 (3)	0.0468 (9)	
C7	0.1002 (3)	0.1766 (4)	0.4281 (3)	0.0467 (9)	
C8	0.0945 (2)	0.2785 (4)	0.5071 (4)	0.0450 (9)	
C9	0.1819 (3)	0.2889 (4)	0.5819 (3)	0.0502 (9)	
C10	0.2475 (4)	0.3995 (6)	0.7344 (4)	0.0867 (14)	
H10A	0.3070	0.4555	0.7042	0.130*	
H10B	0.2706	0.2951	0.7557	0.130*	
H10C	0.2218	0.4579	0.7930	0.130*	
C11	0.0106 (3)	0.5495 (4)	0.5090 (3)	0.0595 (10)	
H11A	0.0695	0.5878	0.5513	0.071*	
H11B	−0.0548	0.6004	0.5331	0.071*	
C12	0.0304 (4)	0.5986 (7)	0.4006 (5)	0.1008 (19)	
H12	0.0732	0.5271	0.3642	0.121*	
C13	0.0036 (6)	0.7061 (11)	0.3530 (7)	0.161 (3)	
H13A	−0.0395	0.7845	0.3826	0.194*	
H13B	0.0249	0.7154	0.2848	0.194*	

### Atomic displacement parameters (Å^2^)

**Table:**

	*U*^11^	*U*^22^	*U*^33^	*U*^12^	*U*^13^	*U*^23^
S1	0.0376 (4)	0.0683 (6)	0.0593 (6)	0.0050 (4)	0.0069 (5)	0.0078 (7)
O1	0.0553 (15)	0.088 (2)	0.076 (2)	−0.0060 (14)	0.0169 (15)	0.0296 (19)
O2	0.0418 (12)	0.0919 (19)	0.080 (2)	0.0220 (12)	0.0017 (16)	−0.0075 (19)
O3	0.0477 (15)	0.0674 (19)	0.086 (3)	0.0147 (14)	−0.0021 (13)	−0.0186 (15)
O4	0.0474 (14)	0.0682 (17)	0.082 (2)	0.0146 (13)	−0.0103 (13)	−0.0130 (18)
O5	0.0537 (14)	0.0741 (19)	0.0557 (19)	0.0121 (13)	−0.0078 (13)	−0.0097 (16)
N1	0.0439 (14)	0.0501 (18)	0.053 (3)	0.0077 (13)	0.0021 (12)	0.0005 (16)
C1	0.046 (2)	0.037 (2)	0.064 (3)	−0.0039 (16)	−0.0024 (18)	0.006 (2)
C2	0.055 (2)	0.055 (3)	0.084 (4)	0.005 (2)	−0.012 (2)	−0.001 (3)
C3	0.074 (3)	0.072 (3)	0.075 (4)	0.001 (2)	−0.025 (2)	−0.010 (3)
C4	0.092 (3)	0.064 (3)	0.060 (3)	−0.005 (2)	−0.011 (3)	−0.016 (2)
C5	0.057 (2)	0.052 (3)	0.072 (4)	−0.0030 (18)	0.001 (2)	−0.013 (2)
C6	0.0473 (19)	0.039 (2)	0.054 (3)	−0.0024 (15)	−0.0026 (18)	−0.0014 (19)
C7	0.0384 (18)	0.043 (2)	0.059 (3)	0.0018 (15)	0.0066 (18)	0.0058 (19)
C8	0.0376 (15)	0.0445 (19)	0.053 (3)	0.0037 (15)	0.005 (2)	0.001 (2)
C9	0.048 (2)	0.046 (2)	0.057 (3)	0.0034 (18)	0.0015 (19)	0.005 (2)
C10	0.077 (2)	0.113 (4)	0.070 (3)	0.024 (3)	−0.031 (2)	−0.027 (3)
C11	0.061 (2)	0.050 (2)	0.068 (3)	0.0065 (17)	−0.002 (2)	0.001 (2)
C12	0.093 (4)	0.070 (4)	0.139 (6)	0.008 (3)	0.010 (3)	0.036 (4)
C13	0.211 (9)	0.131 (7)	0.142 (7)	−0.031 (6)	−0.035 (6)	0.050 (5)

### Geometric parameters (Å, °)

**Table:**

S1—O2	1.423 (2)	C4—C5	1.375 (5)
S1—O1	1.424 (3)	C4—H4	0.9300
S1—N1	1.625 (3)	C5—C6	1.380 (5)
S1—C1	1.746 (4)	C5—H5	0.9300
O3—C7	1.343 (4)	C6—C7	1.462 (5)
O3—H3O	0.8200	C7—C8	1.338 (5)
O4—C9	1.218 (4)	C8—C9	1.461 (5)
O5—C9	1.315 (4)	C10—H10A	0.9600
O5—C10	1.440 (5)	C10—H10B	0.9600
N1—C8	1.438 (4)	C10—H10C	0.9600
N1—C11	1.473 (4)	C11—C12	1.490 (7)
C1—C2	1.390 (5)	C11—H11A	0.9700
C1—C6	1.396 (5)	C11—H11B	0.9700
C2—C3	1.366 (6)	C12—C13	1.142 (8)
C2—H2	0.9300	C12—H12	0.9300
C3—C4	1.380 (6)	C13—H13A	0.9300
C3—H3A	0.9300	C13—H13B	0.9300
			
O2—S1—O1	119.46 (17)	C1—C6—C7	119.4 (3)
O2—S1—N1	108.03 (15)	C8—C7—O3	122.7 (3)
O1—S1—N1	107.87 (16)	C8—C7—C6	123.7 (3)
O2—S1—C1	110.57 (18)	O3—C7—C6	113.5 (3)
O1—S1—C1	107.1 (2)	C7—C8—N1	120.8 (3)
N1—S1—C1	102.48 (15)	C7—C8—C9	120.7 (3)
C7—O3—H3O	109.5	N1—C8—C9	118.4 (3)
C9—O5—C10	116.0 (3)	O4—C9—O5	123.6 (3)
C8—N1—C11	118.1 (3)	O4—C9—C8	121.9 (4)
C8—N1—S1	114.3 (2)	O5—C9—C8	114.4 (3)
C11—N1—S1	120.1 (2)	O5—C10—H10A	109.5
C2—C1—C6	121.4 (4)	O5—C10—H10B	109.5
C2—C1—S1	121.2 (3)	H10A—C10—H10B	109.5
C6—C1—S1	117.5 (3)	O5—C10—H10C	109.5
C3—C2—C1	118.8 (4)	H10A—C10—H10C	109.5
C3—C2—H2	120.6	H10B—C10—H10C	109.5
C1—C2—H2	120.6	N1—C11—C12	113.8 (4)
C2—C3—C4	120.7 (4)	N1—C11—H11A	108.8
C2—C3—H3A	119.7	C12—C11—H11A	108.8
C4—C3—H3A	119.7	N1—C11—H11B	108.8
C5—C4—C3	120.3 (4)	C12—C11—H11B	108.8
C5—C4—H4	119.9	H11A—C11—H11B	107.7
C3—C4—H4	119.9	C13—C12—C11	133.1 (7)
C4—C5—C6	120.7 (4)	C13—C12—H12	113.5
C4—C5—H5	119.7	C11—C12—H12	113.5
C6—C5—H5	119.7	C12—C13—H13A	120.0
C5—C6—C1	118.1 (3)	C12—C13—H13B	120.0
C5—C6—C7	122.4 (3)	H13A—C13—H13B	120.0
			
O2—S1—N1—C8	−167.6 (2)	S1—C1—C6—C7	−2.9 (5)
O1—S1—N1—C8	62.0 (3)	C5—C6—C7—C8	159.4 (4)
C1—S1—N1—C8	−50.8 (3)	C1—C6—C7—C8	−19.9 (6)
O2—S1—N1—C11	−18.4 (4)	C5—C6—C7—O3	−21.1 (5)
O1—S1—N1—C11	−148.8 (3)	C1—C6—C7—O3	159.6 (3)
C1—S1—N1—C11	98.4 (3)	O3—C7—C8—N1	−177.3 (3)
O2—S1—C1—C2	−31.7 (4)	C6—C7—C8—N1	2.2 (6)
O1—S1—C1—C2	100.0 (3)	O3—C7—C8—C9	0.0 (6)
N1—S1—C1—C2	−146.6 (3)	C6—C7—C8—C9	179.5 (3)
O2—S1—C1—C6	149.8 (3)	C11—N1—C8—C7	−112.6 (4)
O1—S1—C1—C6	−78.6 (3)	S1—N1—C8—C7	37.3 (4)
N1—S1—C1—C6	34.8 (3)	C11—N1—C8—C9	70.1 (4)
C6—C1—C2—C3	0.8 (6)	S1—N1—C8—C9	−140.1 (3)
S1—C1—C2—C3	−177.7 (3)	C10—O5—C9—O4	2.4 (5)
C1—C2—C3—C4	−1.0 (6)	C10—O5—C9—C8	−179.0 (3)
C2—C3—C4—C5	1.3 (7)	C7—C8—C9—O4	3.8 (6)
C3—C4—C5—C6	−1.3 (6)	N1—C8—C9—O4	−178.8 (3)
C4—C5—C6—C1	1.1 (6)	C7—C8—C9—O5	−174.8 (3)
C4—C5—C6—C7	−178.2 (4)	N1—C8—C9—O5	2.6 (5)
C2—C1—C6—C5	−0.8 (6)	C8—N1—C11—C12	68.0 (4)
S1—C1—C6—C5	177.7 (3)	S1—N1—C11—C12	−80.1 (4)
C2—C1—C6—C7	178.5 (3)	N1—C11—C12—C13	144.7 (8)

### Hydrogen-bond geometry (Å, °)

**Table:**

*D*—H···*A*	*D*—H	H···*A*	*D*···*A*	*D*—H···*A*
O3—H3O···O4	0.82	1.84	2.555 (4)	146
C11—H11A···O5	0.97	2.50	3.055 (4)	116
C3—H3A···O1^i^	0.93	2.51	3.406 (6)	163

Symmetry codes: (i) −*x*−1/2, *y*, *z*−1/2.
